# Sigmoid ventricular septum treated with endocardial ablation to improve left ventricular outflow: cases report

**DOI:** 10.3389/fcvm.2024.1439504

**Published:** 2024-10-03

**Authors:** Shen Huang, Xiuyu Wang, Qiyan Li, XinLin Xiong, Chuan He, Kun Feng, Jiafa Jing, Jun Ma

**Affiliations:** ^1^ Department of Cardiology, Clinical Medical College & Affiliated Hospital of Chengdu University, Chengdu, Sichuan, China; ^2^ Department of Cardiology, West China Hospital, Sichuan University, Chengdu, Sichuan, China

**Keywords:** radiofrequency ablation, ventricular outflow obstruction, dobutamine, hypertrophic cardiomyopathy (HCM), structural heart disease intervention

## Abstract

**Background:**

Sigmoid Ventricular Septum (SVS) is a type of hypertrophic cardiomyopathy characterized by a reduced angle between the basal interventricular septum and the ascending aorta, and SVS can lead to dynamic Left Ventricular Outflow Tract obstruction (LVOTO) during hypercontractile states. Patients experiencing LVOTO may manifest symptoms such as angina, syncope, etc. Radiofrequency ablation (RFA) has been utilized to treat patients with hypertrophic obstructive cardiomyopathy, but there is no reports on its use in treating LVOTO resulting from SVS. Our report describes two cases of SVS treated with endocardial ablation to improve LVOTO.

**Case report:**

Case 1: A 74-year-old female patient with angina and syncope was admitted to the hospital and diagnosed with SVS by transthoracic echocardiogram. The patient exhibited LVOTO and Systolic Anterior Motion (SAM) phenomena during the administration of the dobutamine stress test. After RFA was performed, the patient's symptoms significantly improved. Additionally, septum decreased from 16 to 13 mm after ten months, and the morphological changes associated with SVS also disappeared. Case 2: A 57-year-old female was admitted to the hospital due to recurrent chest pain after physical activity for more than four years. The transthoracic echocardiogram indicated that the patient met the diagnostic criteria for SVS, and LVOTO and SAM phenomenaoccurred following dobutamine stress test. The patient had significant improvement in symptoms after undergoing RFA treatment.

**Conclusions:**

These two cases represent the first documented instances where dynamic LVOTO caused by SVS could be effectively managed through endocardial RFA.

## Introduction

Sigmoid Ventricular septum (SVS) (1), or Sigmoid-shaped interventricular septum (SIS) (2), is a morphological alteration involving a reduced angle between the basal interventricular septum and the ascending aorta. SVS is closely related to age and peak systolic blood pressure, and it is a cardiovascular risk factor (1, 3).

SVS is a type of hypertrophic cardiomyopathy with a unique structure. The SVS is according to the following criteria: (1) an upper interventricular septal thickness ≥14 mm; (2) an upper septal thickness/mid-septal thickness ratio ≥1.3; (3) an angle between the anterior wall of the aorta and right ventricular side of the interventricular septum (septal *θ*) < 120° in the parasternal long-axis view; and (4) no wall motion abnormalities or mid-septal scarring that could result in isolated septal thickening (1). SVS can lead to clinical symptoms, including angina, syncope etc., due to the occurrence of dynamic left ventricular outflow tract obstruction (LVOTO) (2, 4). Some cases of LVOTO can be alleviated with negative inotropic agents, others show poor responsiveness to such treatment (5). There is currently no widely recognized treatment for SVS. In this report, we present two cases of SVS where successful relief of LVOTO was achieved through endocardial radiofrequency ablation (RFA).

All procedures involving human participants were approved by the ethics committee of Affiliated Hospital of Chengdu University (reference number: PJ2023-064-01), and written informed consent was obtained from the patients and their family before the RFA.

## Case 1

**Figure 1:**
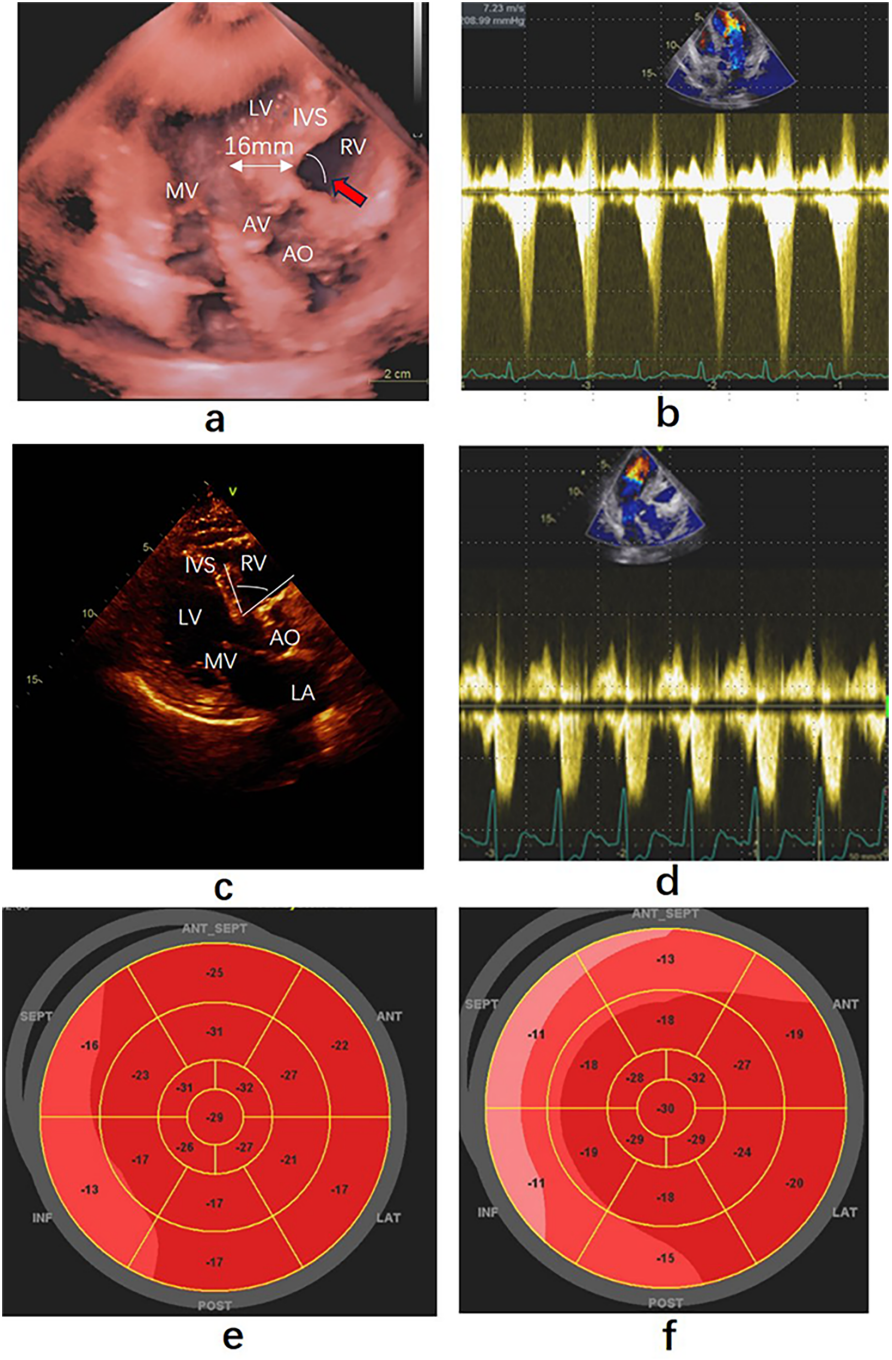
Echocardiography findings before and after precedure. **(a)** Apical three-chamber view showing the thickness of the base ventricular septum was 16 mm, pointed out by the white double arrow. Red arrow point to the angle between the anterior wall of the aorta and the right ventricular side **(b)** Left ventricular outflow gradient provoked by dobutamine before ablation. **(c)** Parasternal long-axis view of echocardiography taken ten months after ablation, the thickness of thebase ventricular septum was 13 mm. White arc was the angle between the anterior wall of the aorta and the right ventricular side. **(d)** Left ventricular outflow gradient provoked by dobutamine after ablation. **(e,f)** Strain bullseye plot illustrating regional longitudinal strain in all myocardial segments before and after ablation. RA, right atrium; RV, right ventricle; LV, left ventricle; LA, left atrium; AO, Aorta; AV, aortic valve; MV, mitral valve. SEPT: Posterior Septum, ANT-SEPT: Anterior Septum.

A 74-year-old Asian female patient was admitted to the hospital due to angina and syncope. She was an obese patient (Body Mass Index, BMI = 31) with hypertension history, currently she was taking a daily regimen of candesartan (4 mg), indapamide (1.5 mg), and bisoprolol (5 mg) to manage her condition. A year ago, she underwent coronary angiography, which revealed no abnormalities. Upon examination, her blood pressure was measured 172/84 mmHg, and her heart rate was 65 bpm, maintaining a regular rhythm. A transthoracic echocardiogram showed normal left ventricular function with a left ventricular ejection fraction of 65%. There was also evidence of upper interventricular septal thickening, particularly in the basal portion of the interventricular septum (IVS), measuring 16 mm (Figure 1a) and protruding into the left ventricular outflow tract (LVOT). The end-diastolic thickness of the mid-septum and LV posterior wall was 10 mm. The ratio of basal thickness to mid-septal thickness was 1.6. Additionally, the angle between the anterior wall of the aorta and the right ventricular side of the interventricular septum was less than 120° (Figure 1a). At rest, her heart rate was 86 bpm, and the maximum blood flow velocity in the LVOT was measured at 1.16 m/s, with a maximum pressure gradient (MaxPG) of 5 mmHg. No signs of SAM of the mitral valve were observed. A dobutamine stress echocardiography was performed to further evaluate the presence of dynamic LVOTO, incrementally increasing the heart rate from 5–20 ug/kg. At a heart rate of 118 bpm, the patient experienced angina and pre-syncope, accompanied by signs of SAM, resulting in LVOTO.During this phase, the continuous Doppler flow velocity at the LVOT increased to 7.23 m/s (Figure 1b) with a MaxPG of 209 mmHg. Since the patient continued to exhibit clinical symptoms despite beta-blocker therapy, a decision was made to proceed with endocardial RFA.

**Figure 2:**
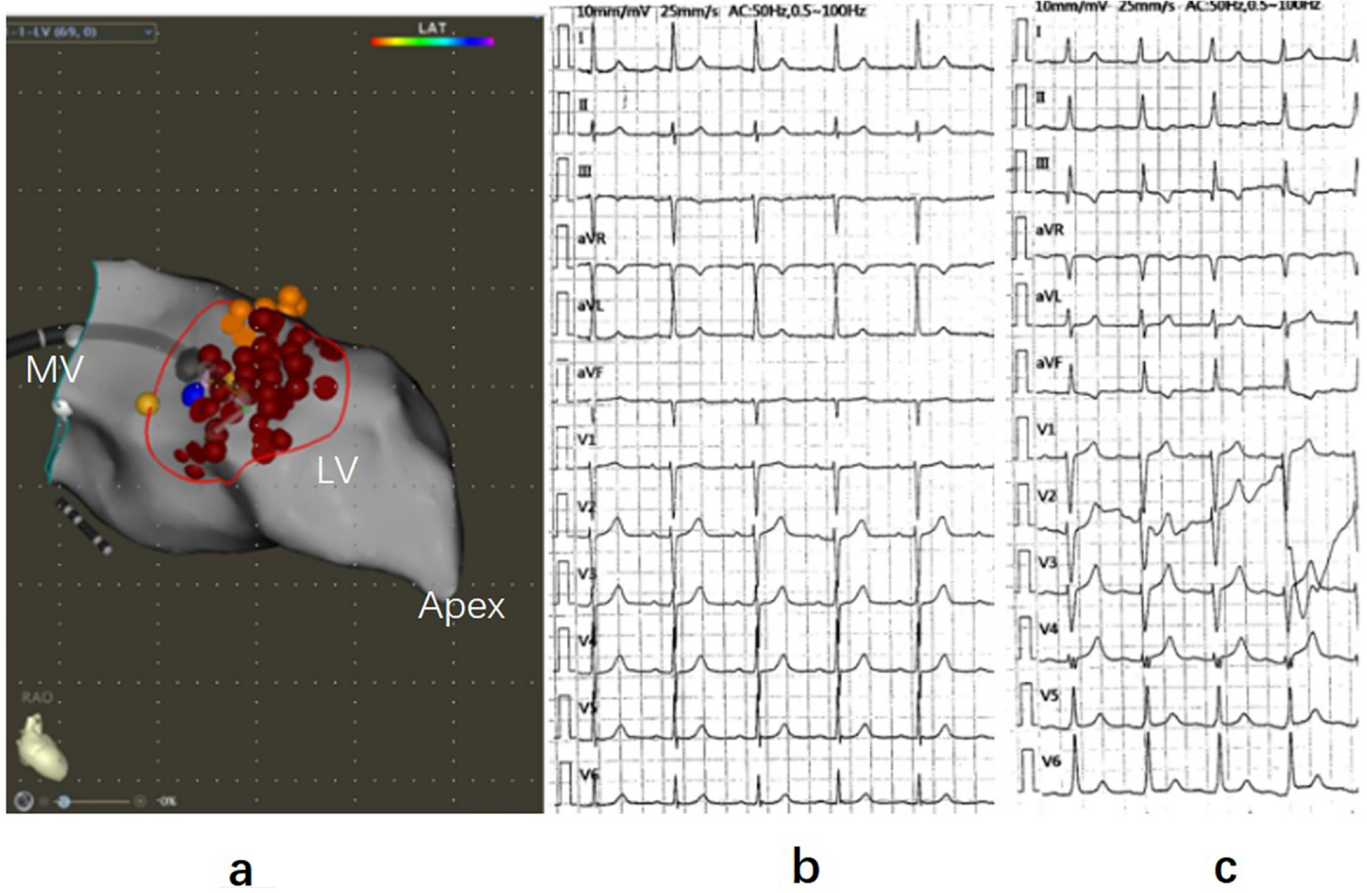
Three-dimensional mapping of ventricular septal ablation and electrocardiogram. **(a)** Left ventricular septal endocardial radiofrequency ablation guided by 3D mapping in the RAO view. Red dots represent ablation points, yellow dots indicate His potential and P potential, and the blue dot marks the thickest part of the ventricular septal base. **(b)** Electrocardiogram (ECG) recorded before ablation. **(c)** ECG during ablation displaying left posterior branch block. RAO, right anterior oblique; LV, left ventricle; MV, mitral valve.

The procedure was performed under general anesthesia. A 4-pole electrode was inserted through the right femoral vein and carefully maneuvered into the right ventricle (RV) for backup pacing. The SL1 sheath was inserted through the right femoral vein to facilitate atrial septal puncture. The patient received heparinization (100 U/kg), with the dosage adjusted to maintain an activated clotting time of 250–300 s. Electroanatomic mapping of the left ventricle was conducted using the CARTO system (Figure 2a). RFA was carried out utilizing a 4-mm, irrigated-tip ablation catheter (Thermocool Smart Touch, Biosense-Webster Inc., Diamond Bar, CA), which was introduced through the SL1 sheath, passing across the mitral annulus and entering the left ventricle. Transesophageal echocardiography guided the catheter to the contact point between the anterior mitral valve lobe and the left ventricular septum for ablation. The ablation was performed at a power setting of 40–45 W, with contact force between 5 and 20 g. Each point receiving 60–90 s of ablation time and a flow rate of 20 ml/min. During the ablation process, the HIS bundle and P potential were carefully identified and avoided. The primary ablation objective was to cover the area of SAM-septal contact and the thickest part of the ventricular septum as much as possible. The ablation was terminated when the electrocardiogram displayed a left posterior branch block pattern (Figures 2b,c), and the MaxPG decreased to 18 mmHg after the dobutamine stress test.

After ablationand five days following with patient, SAM and LVOTO were no longer observed during the dobutamine stress test, with the heart rate reaching 121 bpm. The flow velocity in the LVOT was measured at 2.66 m/s (Figure 1d), and MaxPG was recorded as 28 mmHg. A strain bulls-eye plot illustrated a decrease in regional longitudinal strain (LS) in the SEPT and ANT-SEPT segments after the ablation (before vs. after = −16% vs. −11%) (Figures 1e,f). After ten months of follow-up, the patient's symptoms were alleviated. Transthoracic cardiac ultrasound revealed the disappearance of morphological changes in SVS, with the basal portion of the IVS and the posterior wall thickness at 13 mm (Figure 1c). The angle between the basal interventricular septum and the ascending aorta remained less than 120°.

## Case 2

**Figure 3:**
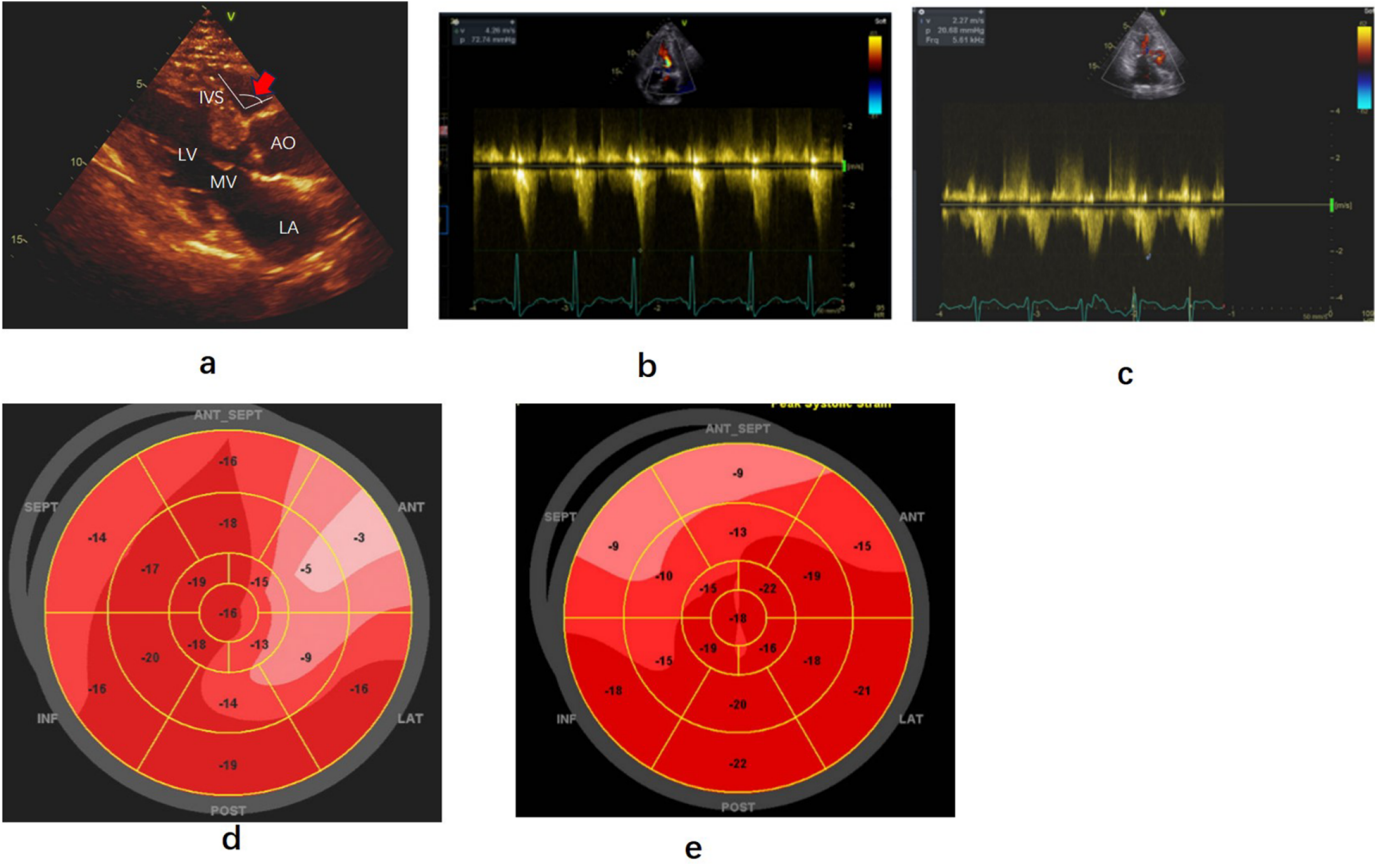
Echocardiography findings before and after precedure. **(a)** Parasternal long-axis view of echocardiography showing hypertrophy of the base of the left ventricular septum and the angle between the aorta and right ventricular interventricular septum(red arrow). **(b,c)** Left ventricular outflow gradient provoked by dobutamine before and after radiofrequency ablation. **(d,e)** Strain bullseye plotsduring baselineand three months after dobutamine stress post-ablation. RA, right atrium; RV, right ventricle; LV, left ventricle; LA, left atrium; AO, Aorta; AV, aortic valve; MV, mitral valve; SEPT, posterior septum; ANT-SEPT, anterior septum.

A 57-year-old Asian female, with a BMI of 28, was admitted to the hospital due to recurrent chest pain, fatigue, and shortness of breath after physical activity. She had a 34-year history of hypertension, with systolic pressures exceeding 180 mmHg at times. She was on a daily regimen of amlodipine (5 mg) and indapamide (1.5 mg), effectively controlling her blood pressure. Coronary angiography revealed mid-median stenosis with calcification in the left anteriorproximal descending branch, while the other coronary branches appeared normal. A 2/6 grademurmur was detected at the left sternal border in the third to fourth intercostal space during auscultation. Transthoracic echocardiography revealed subaortic ventricular septal thickening (approximately 17 mm) and normal left ventricular function, indicated by an ejection fraction of 56%. SAM with LVOTO was observed. The end-diastolic thickness of the mid-septum and LV posterior wall was 12 mm, with a basal-to-median thickness ratio of 1.42. Furthermore, the angle between the anterior wall of the aorta and the right ventricular side of the interventricular septum was less than 120° (Figure 3a).

**Figure 4:**
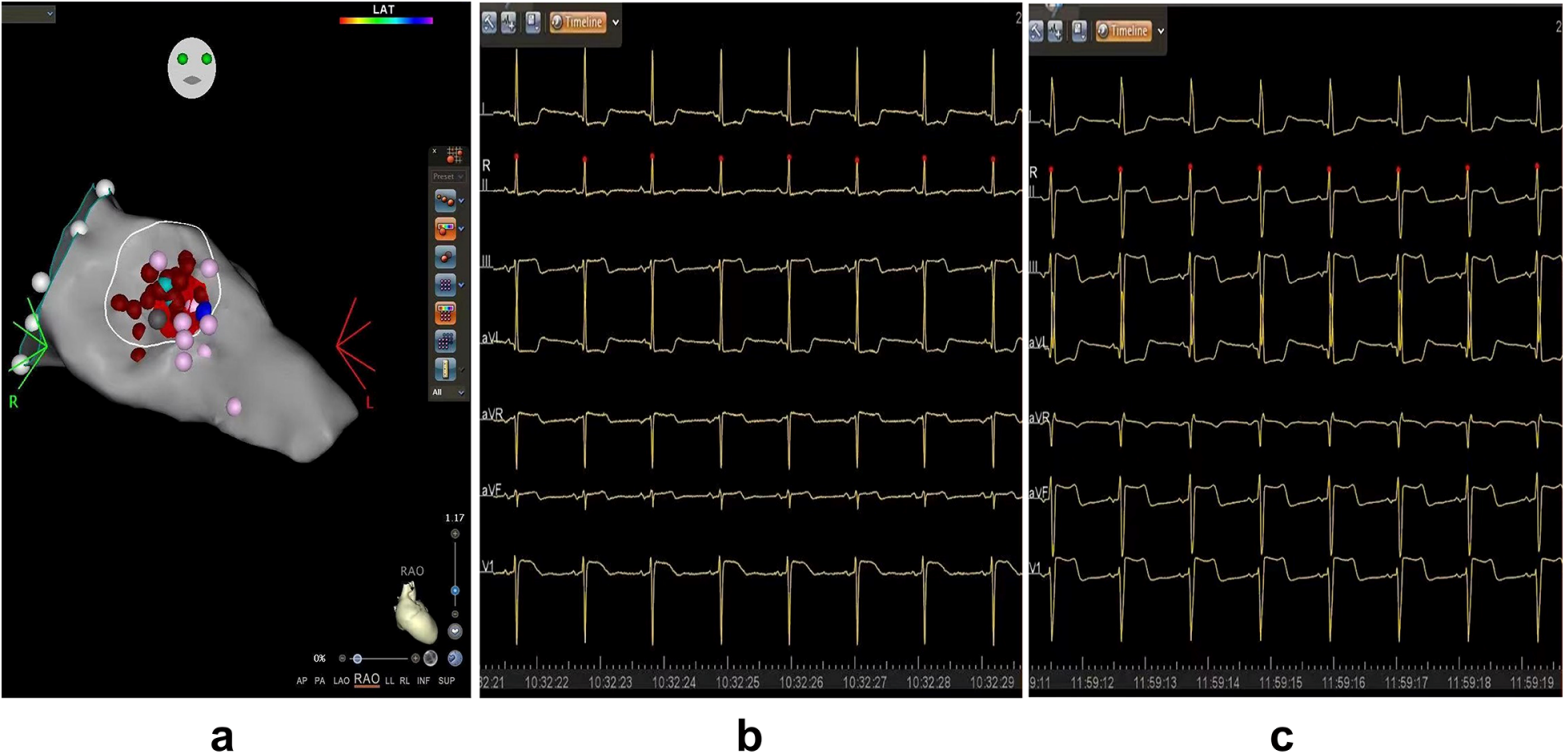
Three-Dimensional mapping of ventricular septal ablation and electrocardiogram. **(a)** Left ventricular septal endocardial radiofrequency ablation in the RAO view. Red dots represent ablation points, purple points indicate His potential and P potential, and light blue dot marks the thickest part of the ventricular septal base. **(b)** ECG recorded before ablation, demonstrating a normal electric axis with ST-segment depression in leads I and aVL. **(c)** ECG during ablation indicating left axis deviation with ST-segment depression in lead I, aVL, and slight elevation in lead II, III, aVF, and V1. LV, left ventricle; MV, mitral valve.

At rest, the blood flow velocity in the left ventricular outflow tract was 3.02 m/s, with a MaxPG of 36 mmHg. Following dobutamine infusion (55 ug/kg/min), when the heart rate reached 95 bpm, the SAM phenomenon worsened, resulting in a blood flow velocity in the LVOT of 4.26 m/s and a MaxPG of 73 mmHg (Figure 3b). Despite being on bisoprolol (5 mg daily) to control heart rate for several months, the patient reported only modest relief from her symptoms of shortness of breath and chest pain. Consequently, she underwent ventricular septal RFA treatment under general anesthesia (Figure 4a). The procedure was similar tothat performed in Case 1, with ablation ceasing upon the appearance of a left anterior branch block on the electrocardiogram (Figures 4b,c). Immediately after ablation, dobutamine was administered (30 ug/kg/min), resulting in a notable improvement in the mitral valve SAM phenomenon, with a MaxPG of 27 mmHg and a heart rate of 110 bpm. Her symptoms significantly improved during the follow-up period. Three months later, a dobutamine stress test (30 ug/kg/min) was conducted, with the heart rate reaching 109 bpm. The LVOT pressure gradient decreased significantly, and the blood flow velocity measured 2.27 m/s, with a MaxPG of 21 mmHg (Figure 3c). A strain bulls-eye plot revealed a reduction in regional longitudinal strain in the anterior segments after ablation during dobutamine stress (LS −9% vs. −6%) (Figures 3d,e). The thickness of the base of the ventricular septum remained at approximately 17 mm. The angle between the basal interventricular septum and the ascending aorta remained less than 120°.

## Discussion

In this study, we propose a novel approach using RFA for the treatment of SVS. To our knowledge, this is the first report of such a method being applied to SVS treatment.

The treatment methods for SVS are under debate, drug therapy (including β-blockers, Cibenzoline etc.) requires long-term use, and the effects are sometimes not ideal (2, 6). Myectomy of the subaortic hypertrophied septal bulge may lead to a complete atrioventricular block andiatrogenic ventricular septal defect (7). Percutaneous transluminal septal myocardial ablation with absolute ethanol has been successfully employed for symptomatic SVS (8), up to 10% of patients may not be suitable for alcohol septal ablation due to the absence of a target septal perforator or the presence of a perforator supplying other myocardial regions (9). Furthermore, there is a risk that the mid-septal thickness of SVS may not be sufficient to prevent perforation of the interventricular septum.

In both of our cases, RFA was used for the treatment of SVS. Dobutamine-induced left ventricular MaxPG significantly decreased following ablation, along with the disappearance or improvement of the SAM phenomenon. Ventricular septal RFA for treating outflow tract obstruction in hypertrophic obstructive cardiomyopathy was initially reported by Emmel and Sreeram (10). In this study, we applied RFA to SVS, and the mechanism of RFA in reducing left ventricular MaxPG in hypertrophic obstructive cardiomyopathy is similar. LS is used to assess the longitudinal contractile capacity of the subendocardial myocardium, with more negative LS indicating better local myocardial segmental motor function (11, 12). A strain bulls-eye plot in the first patientindicated a reduction in regional LS in the septum and anteroseptum segments after ablation (−16% vs. −11%). In the second patient, longitudinal strain in the anterior segment decreased after ablation duringdobutamine stress (−9% vs. −6%). These findings suggest local myocardial contractile dysfunction, which likely reduces the outflow tract pressure gradient.

As an invasive treatment, the main risks and limitations of RFA are related to the procedure itself and the preoperative condition of the patient. If the patient cannot tolerate surgery [surgical conditions refer to hypertrophic cardiomyopathy ablation (13)], this treatment method should not be used. Additionally, the risks associated with the invasive procedure, such as bleeding and cardiac rupture, must be considered. Therefore, preoperative evaluation and obtaining informed consent from the patient are very important. Besides, in our cases, both patients exhibited damaged left bundle branches, with one presenting with a left posterior fascicular block and the other with a left anterior fascicular block. The left bundle branch courses directly beneath the target area of SAM-septal contact, making it inevitable that ablating this area may damage the conduction tissue. However, animal experiments confirmed that LV remodeling occurs after RFA damages the left bundle branch in dogs (14). Whether left bundle branch block after ablation is a prerequisite for achieving therapeutic efficacy or an adverse side effect of the surgery still requires more clinical research to be confirmed.

It is worth mentioning that several new ablation modalities have recently been applied in cardiac ablation procedures, such as temperature-controlled RFA, pulsed field ablation, and radiofrequency needle ablation (15–17). These methods have not only demonstrated good efficacy but also significantly reduced the risk of steam pops, thereby greatly lowering surgical risk. These novel ablation techniques may offer new treatment options for SVS.

## Conclusions

In summary, these two cases provide the first evidence that dynamic left ventricular outflow tract obstruction caused by SVS can be effectively treated with endocardial RFA. Although the two cases may not represent a universal response among all patients with SVS, they suggest a potential method for clinicians managing this condition. The effectiveness and safety of this procedure require further validation in future studies.

## Data Availability

The raw data supporting the conclusions of this article will be made available by the authors, without undue reservation.
